# Protecting Healthcare Professionals during the COVID-19 Pandemic

**DOI:** 10.1155/2020/8469560

**Published:** 2020-10-05

**Authors:** Baiwen Qi, Haiheng Peng, Kangquan Shou, Zhengyu Pan, Min Zhou, Rui Li, Liping Deng, Jun Shen, Xin Rao, Aixi Yu

**Affiliations:** ^1^Department of Orthopedics Trauma and Microsurgery, Zhongnan Hospital, Wuhan University, Wuhan, 430071 Hubei, China; ^2^Department of Orthopedic, The First College of Clinical Medical Science, China Three Gorges University& Yichang Central People's Hospital, Yichang, Hubei, China; ^3^Department of Healthcare Management, School of Health Sciences, Global Health Institute, Wuhan University, China; ^4^Department of Infectious Disease, Zhongnan Hospital of Wuhan University, Wuhan, Hubei, China; ^5^Department of Emergency Medicine, Zhongnan Hospital of Wuhan University, Wuhan, Hubei, China; ^6^Department of Critical Care Medicine, Zhongnan Hospital of Wuhan University, Wuhan, Hubei, China

## Abstract

**Objective:**

To understand how to implement proactive prevention measures among healthcare professionals for preventing potential nosocomial infection.

**Methods:**

91 healthcare professionals confirmed with the COVID-19 infection were collected, and clinical characteristics and epidemiological data were evaluated.

**Results:**

Among the cases, 77 cases (84.6%) were confirmed by the viral nucleic acid test, and the other 14 cases were diagnosed by the clinical investigation. Ground glass opacity and bilateral shadows distribution were observed in 78 cases (85.6%). 56 cases (61.5%) were admitted into Zhongnan Hospital and subjected to antiviral treatment. 73 of a total of 91 cases (80.2%) with a median incubation period of 3 days (IQR, 2 to 6) reported close contact history with patients with the COVID-19 infection. The most common symptoms at the onset of illness were fever (66 cases, 72.5%) and cough (54 cases, 59.3%). The initial positive rate of the CT scan and RT-PCR assay were 84.6% and 48.4%, respectively (*P* < 0.01). There were 50 cases occurred during the early stage (before Jan 20, 2020), whereas 41 cases occurred at a late stage (after Jan 20, 2020). In the early stage, the most common route of exposure to COVID-19 was via direct care in the absence of any invasive procedure. By contrast, 37 healthcare professionals infected with COVID-19 in the late stage were confirmed to have been exposed via aerosol-generating procedures.

**Conclusion:**

Identification of the asymptomatic individuals in healthcare settings and prompt response when a suspicious case is considered may render effective control of the nosocomial infection during this pandemic.

## 1. Introduction

With the rapid escalation of the infected number of patients with the coronavirus disease 2019 (COVID-19), there is a growing concern of infection among healthcare professionals. Previously, a total of 3387 healthcare professionals in China were diagnosed with COVID-19, as the infectious disease has rapidly spread throughout the whole country [1]. As noted, healthcare professionals are at the front line of the COVID-19 outbreak response and as such are exposed to hazards (pathogen exposure, psychological distress, long working hours, etc.) that put them at risk of infection. Therefore, the safety of healthcare professionals is a major challenge for all healthcare settings around the world, and missteps may have serious health implications for healthcare professionals.

Unfortunately, most of the patients with high risk of transmission were proven to be asymptomatic in the early stage [2]. The nonspecific signs or symptoms of COVID-19 may give rise to two significant challenges, not only for the early identification and further isolation of infectious patients but also for prevention from secondary transmission within the healthcare setting and environment, particularly from patients to healthcare professionals. More importantly, the optimal personal protection equipment (PPE) remains unclear so far because it has been reported that healthcare professionals were confirmed to have infection of COVID-19 even though they were implementing the superior precautionary measures by wearing N95 masks and visors, as well as gowns [3]. Therefore, how to utilize PPE appropriately is critical for healthcare professionals because of their indispensable roles in the clinical management system, thereby assuring the prevention of future nosocomial infection at the global scale.

In this retrospective study, we aimed to describe and investigate transmission characteristics among healthcare professionals confirmed to have the COVID-19 infection and to compare the infection features among these cases before and after the implementation of upgraded personal protection as well as the modified precautionary strategy. Our report may be helpful for a comprehensive understanding of COVID-19 transmission among healthcare professionals and the role of the implementation of advanced personal infection-prevention measures for reducing nosocomial outbreaks of COVID-19 in healthcare settings.

## 2. Methods

### 2.1. Study Design and Participants

This case series was approved by the institutional ethics board of Zhongnan Hospital of Wuhan University (No. 2020043). For this retrospective, single-center study, we enrolled the healthcare professionals diagnosed with COVID-19 from January 13 to March 23, 2020 at the Zhongnan Hospital of Wuhan University, which is an official assigned hospital for the admission of patients infected with COVID-19. All the cases were diagnosed and enrolled in this study according to the previous reports [4]. The written informed consent was obtained from all the involved cases before data collection.

### 2.2. Procedures and Data Collection

First, we obtained epidemiological, transmission history, clinical, laboratory, treatment, and outcome data of all cases. We further recorded their previous contact/exposure history with confirmed COVID-19 patients basing on the cases who were able to recall the detailed information. The incubation period was defined as the time from exposure to the onset of illness. Next, we enquired about the protection measures and grade [5] as well as the details of the exposure situation. As the official announcement of the confirmed human-to-human potential was declared on January 20, 2020, the comparison of infection features between the cases showing the onset of illness before and after this date were assessed as well. Moreover, we investigated the signs and symptoms on admission as well as the treatment and clinical outcomes, which were followed up to March 31, 2020.

The laboratory investigation of COVID-19 was performed in the Chinese CDC. Particularly, COVID-19 was identified by real-time RT-PCR using the protocol reported previously [4]. Further investigations consisted of the complete blood count and serum biochemical test. All patients were examined by chest CT.

### 2.3. Statistical Analysis

SPSS (version 26.0) was used for all analyses. The median (IQR) was used to describe the continuous measurements if they are not normally distributed, and categorical variables were presented as count (%). Proportions for categorical variables were compared using McNemar's chi-squared test. For unadjusted comparisons, a two-sided *α* of less than 0.05 was considered statistically significant.

## 3. Results

### 3.1. Demographic and Clinic Characteristics

In our baseline scenario, among the 91 COVID-19 confirmed healthcare professionals working in the Zhongnan Hospital of Wuhan University, the median age was 37.0 years (interquartile range 30.0-46.0) with 34 males (37.4%) and 57 females (62.6%). 77 cases (84.6%) were confirmed by the viral nucleic acid test, and the other 14 cases were diagnosed by the clinical investigation according to the revised diagnostic criteria [6]. There were 50 doctors (54.9%) and 38 nurses (41.8%), as well as three medical staff (3.3%). Among them, four cases (4.3%) were accompanied with previous comorbidities (cardiovascular disease, diabetes, and hepatitis). 56 cases (61.5%) required hospital admission, whereas 35 cases (38.5%) were treated as outpatients (Table 1).

73 cases (80.2%) confirmed close contact history with patients diagnosed with COVID-19, whereas other 18 cases (19.8%) denied this. Particularly, the personal protection grade was upgraded after January 20, 2020 when the human-to-human transmission potential was confirmed. The degree of severity of COVID-19 was categorized as nonsevere (uncomplicated illness and mild pneumonia) in 87 cases (95.6%) and severe in four cases (three of them were transferred into the ICU for more advanced respiratory support). There were 50 cases (47 nonsevere cases and three severe cases) occurred at the early stage (before January 20, 2020), whereas 41 cases (40 nonsevere cases, one severe case) occurred at the late stage (after January 20, 2020). In the early stage, the most common route of exposure to COVID-19 was via direct care in the absence of the invasive procedure. By contrast, 37 healthcare professionals infected with COVID-19 in the late stage were confirmed to have been exposed via aerosol-generating procedures including airway suction, intubation, manual ventilation, and cardiopulmonary resuscitation (CPR) (Table 2).

More importantly, the infected cases were variable among different protection grades. In the early stage, 43 cases were infected under no protection measures and six cases with grade I as well as one case with grade II. After the upgraded personal protection measures were conducted, the infected spectrum changed to no infected cases under no particular protection measures and 30 cases with grade I as well as 11 cases with grade II (Table 2). Notably, there were still five cases with infection of COVID-19 under grade III, which was previously considered to be safe precaution measures.

**Figure 1:**
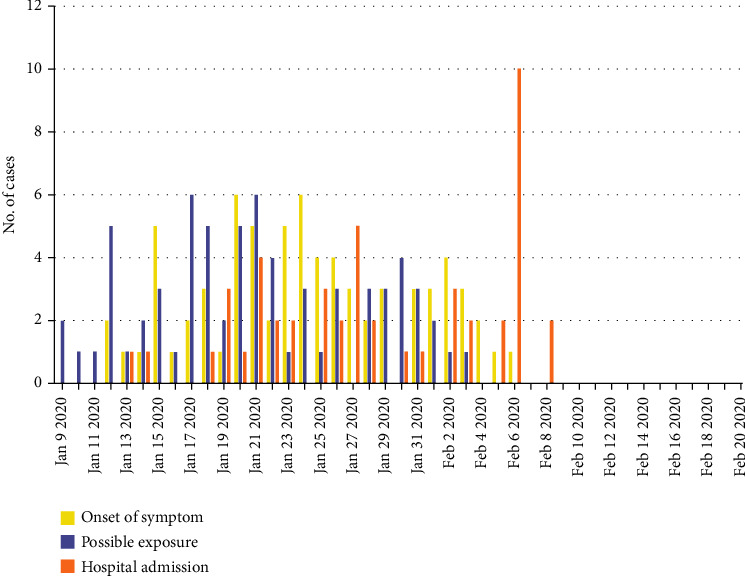
The epidemic curve of COVID-19 among health care professionals from the Zhongnan Hospital of Wuhan University. The numbers of possible COVID-19 infections were speculative, according to the median incubation period calculated in our study.

Among the 56 cases admitted to the hospital, based on the median incubation period calculated as 3 days (interquartile range, 2 to 6), the outbreak of admission occurred in late January (30 cases, 53.6%). 24 cases (42.9%) was traced as the onset of symptom between January 20 and January 27, 2020 (Figure 1).

The most common symptoms were fever (66 cases, 72.5%) and cough (54 cases, 59.3%) as well as myalgia or fatigue (17 cases, 18.7%). Diarrhea (1 case, 1.1%) and palpitation (1 case, 1.1%) were uncommon. The median body temperature was 37.8 (IQR, 37.4-38.4). By using RT-PCR tests, 77 cases (84.6%) were confirmed strongly positive for COVID-19, and five cases (5.5%) were diagnosed as weak positive. 11 cases were diagnosed as COVID-19 basing on the combinatory assessment consisting of clinical symptoms (fever and respiratory tract symptom), decreased or normal white blood cell count, and decreased lymphocyte count as well as hallmark characteristic (ground-glass opacity) in the chest CT scan according to the new coronavirus pneumonia diagnosis and treatment program [6]. On admission, lymphocytopenia was found in 64.8% (59 cases) and leukopenia in 47.3% (43 cases). Notably, the cases with severe pneumonia were more likely to have abnormal laboratory results (including lymphocytopenia and leukopenia) than those with the nonsevere disease (Table 3).

**Figure 2:**
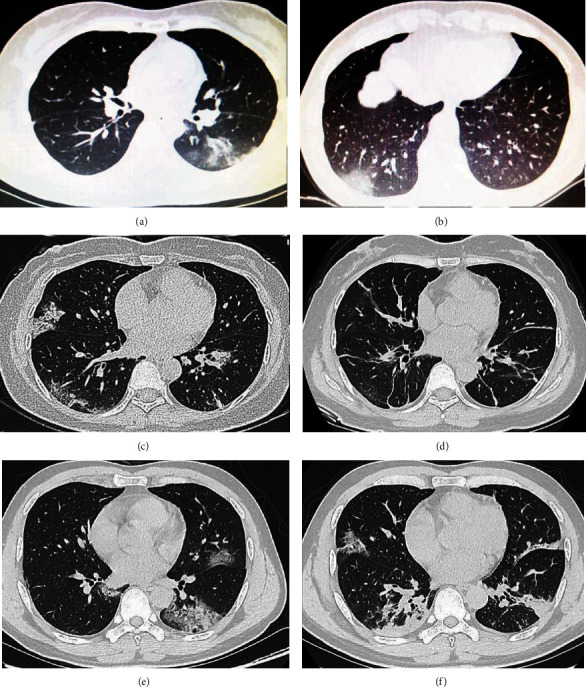
Chest computed tomographic images of three cases infected with COVID-19. (a, b) Computed tomography images of case A obtained on the onset of illness and the admission, respectively. (c, d) Computed tomography images of case B taken before and after the treatment, respectively. (e, f) Computed tomography images of case C indicated the development of the pneumonia.

The most common finding on chest CT was ground-glass opacity or bilateral patchy shadowing (85.6%). Of note, at the initial investigation, 77 cases (84.6%) were confirmed as positive in the chest CT scan, whereas 44 cases (48.4%) were confirmed as positive in the RT-PCR assay. There was a significant difference regarding to the positive finding between the CT scan and RT-PCR assay (*P* < 0.01), indicating a greater degree of diagnostic reliability with the CT scan. The typical bilateral shadows and ground glass opacity in the CT scan were shown as in Figure 2.

### 3.2. Treatment and Outcome

None of the 91 patients were lost to follow-up in our study. As of March 23, 2020, among these 56 cases on admission, five cases (8.9%) were still hospitalized. A total of 51 cases (91.1%) had been discharged, and no fatality was reported. The median days from the onset of symptom to the hospital admission was 4.0 (IQR, 2.0–7.0) (Table 4).

On admission, all the cases were treated with antiviral treatment (interferon alpha inhalation, lopinavir/ritonavir, arbidol, oseltamivir). A large proportion of the patients (47 cases, 83.9%) received intravenous antibiotic therapy. Glucocorticoid therapy was provided for nine patients (16.1%). 39 cases (69.6%) were subjected to the traditional Chinese medicine. 24 cases (42.9%) received adjuvant therapy including hymopeptide and immunoglobulin (Table 4).

## 4. Discussion

Little is known about the characteristics and transmission routes of the ongoing novel coronavirus, namely, COVID-19 [7, 8]. Of particular note, information regarding the optimized precaution policy and measures is scarce, especially in the early stage [9]. Before the confirmation of the human-to-human potential of COVID-19, healthcare professionals were unaware of the risk existing in the routine contact with confirmed COVID-19 patients [10]. This is very similar to the renowned infectious outbreak caused by another coronavirus 17 years ago: more than 20% of cases reported in the 2003 outbreak of severe acute respiratory syndrome (SARS) were healthcare professionals [11, 12]. To protect healthcare professionals from the infectious disease is paramount not only in constituting the front line against high-threat pathogens but also in reducing secondary transmission in the healthcare system [13]. Previously, Xu et al. [14] reported the positive RT-PCR test results of four medical staffs who have been discharged from the hospital, but the author did not evaluate the potential infection routes of these cases. Our findings clearly indicate that advanced precautions policy should be implemented to ensure appropriate infection prevention especially during invasive procedures, and identification of the asymptomatic individuals in the healthcare settings and prompt response when the suspicious case is considered may render effective control of the nosocomial infection during this pandemic.

For this retrospective, single-center study, we enrolled healthcare professionals diagnosed as COVID-19 from January 13 to March 23 2020 at the Zhongnan Hospital of Wuhan University. Compared with the average age of reported cases [15], the median age of infected healthcare professionals in our study was younger (37.0 years). More importantly, the cases in our study were more likely to be nonsevere, showing higher rates of survival and more favorable outcomes. Compared with normal patients, the infected healthcare professionals are more likely to seek medical consultation in the early stage because of their higher professional background.

Based on the discrepancy regarding the infected numbers between the early stage (before January 20) and late stage (after January 20), we conclude that the risk factors for infection of COVID-19 in healthcare professionals could be attributed to the lack of awareness when the disease first emerged, leading to poor institutional infection control policy and lack of the timely controlling of the movement of asymptomatic patients. Furthermore, inappropriate utilization of personal protection equipment (PPE) and frequent exposure to high-risk medical treatments, especially invasive mechanical ventilation, may contribute to the COVID-19 infection of healthcare professionals. More importantly, the major difference between COVID-19 and SARS-CoV in 2003 is the fact that the SARS virus transmission occurs with a lag behind the onset of symptoms [16]. On the contrary, COVID-19 would be more dangerous for healthcare professionals as its transmission ability may occur far ahead of the onset of symptoms [12]. Following the close contact and exposure with the asymptomatic patients, a number of the healthcare professionals were confirmed with the COVID-19 infection in the end of January. Additionally, a large proportion of cases in our study were unaware of the risk arising from the contact with patients initially diagnosed with other diseases instead of COVID-19. Previously, the inadequate personal protection equipment (PPE) was considered as the major reason for the rapid escalation of infection among healthcare professionals in the early stage [17]. However, our results indicated that N95 masks and visors as well as the gowns were not safe enough for preventing infection of COVID-19 because aerosol-generating procedures, such as tracheal intubation and manual ventilation, have been proved to be associated with an increased risk of transmission [4]. Therefore, we strongly recommend that advanced PPE such as positive pressure respirator or helmet should be implemented for healthcare professionals when they perform aerosol-generating procedures. Further contributing to infection among healthcare professionals could be attributed to the floating aerosol with a high concentration in a limited space. Moreover, the previous health condition of the healthcare professionals cannot be ignored as well.

Proactive steps will surely be needed. In our hospital, when a healthcare individual was suspected as infected with COVID-19, the colleagues who had close contact history with this case were quarantined immediately, although the RT-PCR results were not yet obtained. In addition, in this report, we proved that the chest CT exhibited better initial sensitivity than the RT-PCR assay (*P* < 0.01), which is consistent with the previous report [18]. We speculate that this disparity may be attributed to the specimen being collected from the upper respiratory tract instead of the lower respiratory tract. Moreover, repeated RT-PCR tests need to be considered to reduce the false-negative results as well.

The current situation is unprecedented, and it is unclear how long this global pandemic will last. Although there have been reported several studies regarding the transfection among the medical staff [19–23], strict quarantine and provision of prompt treatment still need to be assured for the control of the COVID-19 nosocomial infection. Better education background, earlier self-diagnosis, and more rational psychological status render the healthcare professionals a more favorable outcome during this pandemic. Further efforts to prevent and manage COVID-19 among healthcare professionals still need to be optimized. These strategies need to meet several criteria as follows: (1) ensure early identification of the asymptomatic patients with COVID-19 and comprehensive understanding of its detailed transmission mechanisms in healthcare settings; (2) maintain the compliance with the use of appropriate personal protection equipment (PPE) with top grade; (3) optimize and reorganize the layout of general wards for better triage of patients; (4) and carry out the active and passive quarantine of healthcare professionals as early as possible.

This study has several limitations. First, only 91 patients were included. We are still working on a larger scale study; thus, a more complete epidemiological feature of COVID-19 among healthcare professionals in this outbreak would be identified soon. Second, the median incubation period estimated in our study was calculated based on the memories of the cases; therefore, the uncertainty of the exact detailed information, named recall bias, would be inevitable. As a next step, we are figuring out a more reliable strategy by assessing electronic files, e.g., surveillance-recorded evidence. Third, given the unique feature of this epidemic disease, the onset of symptoms of the suspected case always lags behind the exposure or contact with the identified patients, resulting in the difficulty for estimating the authentic figures of cases who have being infected before January 20, 2020. Thus, more comprehensive epidemic evaluation should be applied.

## 5. Conclusions

The nosocomial outbreak among the healthcare professionals may be the result of unawareness of transmission risk at the onset of the epidemic and low-grade precaution measures. Caution needs to be advised for all hospitals and institutions that an advanced precautions policy includes positive pressure respirator or helmet. Identification of asymptomatic individuals in healthcare settings and a prompt response when a suspicious case is considered may render favorable control of the nosocomial infection during this pandemic.

## Figures and Tables

**Table 1:** Demographics and baseline characteristics of cases with COVID-19.

Characteristics	No. (%), Total (*N* = 91)
Age, median (IQR), yr	37.0 (30.0–46.0)
Sex	
Male	34 (37.4%)
Female	57 (62.6%)
Occupation	
Doctor	50 (54.9%)
Nurse	38 (41.8%)
Others^a^	3 (3.3%)
Chronic medical illness	
Hypertension	2 (2.2%)
Diabetes	1 (1.1%)
HBV(hepatitis B)	1 (1.1%)
Diagnostic method	
RT-PCR assay	77 (84.6%)
Chest CT scan	14 (15.4%)
Treatment	
Outpatient	35 (38.5%)
Hospitalization	56 (61.5%)

^a^Others: laboratory technicians, emergency medical personnel; percentages may not have a total of 100 because of rounding.

**Table 2:** Epidemiological and transmission characteristics of the COVID-19 infection among healthcare professionals.

Characteristics	COVID-19 infection cases, no. (%), total (*N* = 91)		
	Before Jan 20 (*N* = 50)	After Jan 20 (*N* = 41)	Total
Occupation			
Doctor	27 (54.0%)	20 (48.8%)	47 (51.6%)
Nurse	17 (34.0%)	14 (34.1%)	31 (34.1%)
Others^a^	6 (12.0%)	7 (17.1%)	13 (14.3%)
Clinical classifications			
Uncomplicated illness	15 (30.0%)	14 (34.1%)	29 (31.9%)
Mild	32 (64.0%)	26 (63.4%)	58 (63.7%)
Severe	3 (6.0%)	1 (2.4%)	4 (4.4%)
Direct contact history with patients			
Confirmed	36 (72.0%)	37 (90.2%)	73 (80.2%)
Negative	14 (28.0%)	4 (8.8%)	18 (19.8%)
Types of exposure situation			
Direct care without aerosol-generating procedure	28 (56.0%)	4 (9.8%)	32 (35.2%)
Airway suction	12 (24.0%)	9 (22.0%)	21 (23.1%)
Intubation	5 (10.0%)	10 (24.4%)	15 (16.5%)
Manual ventilation	4 (8.0%)	15 (36.6%)	19 (20.9%)
Cardiopulmonary resuscitation	1 (2.0%)	3 (7.3%)	4 (4.4%)
Protection grade			
No protective measure	43 (86.0%)	0	43 (47.3%)
Grade I	6 (12.0%)	30 (73.2%)	36 (39.6%)
Grade II	1 (2.0%)	6 (14.6%)	7 (17.1%)
Grade III	0	5 (12.2%)	5 (12.2%)

^a^Others: laboratory technicians, emergency medical personnel.

**Table 3:** Laboratory findings and radiographic characteristics of cases with COVID-19.

Characteristics	No. (%), total (*N* = 91)
Median incubation period (IQR, days)	3.0 (2.0-6.0)
Median body temperature (IQR, °C)	37.8 (37.4-38.4)
Signs and symptoms	
Fever	66 (72.5%)
Cough	54 (59.3%)
Fatigue	17 (18.7%)
Diarrhoea	1 (1.4%)
Palpitation	1 (1.4%)
CT findings	
Ground-glass opacity or bilateral patchy shadowing	78 (85.6%)
Bilateral pneumonia	8 (8.8%)
Unilateral pneumonia	2 (2.2%)
Pulmonary nodule	1 (1.1%)
No obvious abnormalities	2 (2.2%)
Initial positive rate	77 (84.6%)^∗^
RT-PCR assay	
Positive	77 (84.6%)
Weakly positive	5 (5.5%)
Suspected	6 (6.6%)
Negative	3 (3.3%)
Initial positive rate	44 (48.4%)
Laboratory results	
Leucocytes count (×10^9^/L, normal range 3.5–9.5)	
Increased	0
Normal	48 (52.7%)
Decreased	43 (47.3%)
Lymphocytes count (×10^9^/L, normal range 1.1-3.2)	
Increased	0
Normal	32 (35.1%)
Decreased	59 (64.8%)

Abbreviations: IQR: interquartile range; CT: computed tomography; RT-PCR: real-time reverse transcriptase polymerase chain reaction. Percentages may not have a total of 100 because of rounding. Compared with the initial positive rate of RT-PCR, ^∗^*P* < 0.01.

**Table 4:** Treatment and outcome of cases infected with COVID-19.

Time from onset to admission, days (IQR)	4.0(2.0–7.0)
Treatment	No. (%), total (*N* = 56)
Antiviral treatment	47 (83.9%)
Glucocorticoids	9 (16.1%)
Traditional Chinese medicine	39 (69.6%)
Adjuvant therapy^a^	24 (42.9%)
Outcome	No. (%), total (*N* = 56)
Discharged	51 (91.1%)
Remained in hospital	5 (8.9%)
Died	0 (0%)
Hospital stays, days (IQR) (*n* = 40)	12.0 (8.0-16.0)
Distribution of hospital stays	No. (%), total (*N* = 51)
1~5	3 (5.3%)
6~10	21 (37.5%)
11~15	16 (28.6%)
16~20	9 (16.1%)
21~25	1 (1.8%)
25~28	1 (1.8%)

Abbreviations: IQR: interquartile range; ^a^Adjuvant therapy: thymopeptide or immunoglobulin. Percentages may not have a total of 100 because of rounding.

## Data Availability

The data used to support the findings of this study are available from the corresponding author upon request.
